# Supplementary material for: MetaWise: Combined Feature Selection and Weighting Method to Link the Serum Metabolome to Treatment Response and Survival in Glioblastoma
Source: Int J Mol Sci. 2024 Oct 11;25(20):10965. doi: 10.3390/ijms252010965 (PMC11507606; doi:10.3390/ijms252010965)
Supplement: Supplementary file 1 [file ijms-25-10965-s001.zip › ijms-3242650-supplementary.pdf]

Article

MetaWise: Combined Feature Selection and Weighting Method to Link the Serum Metabolome to Treatment Response and Survival in Glioblastoma

Tasci E [1], Popa M [1], Zhuge Y [1], Chappidi S [1], Zhang L [1], Cooley Zgela T [1], Sproull M [1], Mackey M [1] and

Kates H [2], Garrett TJ [2], Camphausen K [1] and Krauze AV [1]*.

Supplementary: Supplementary

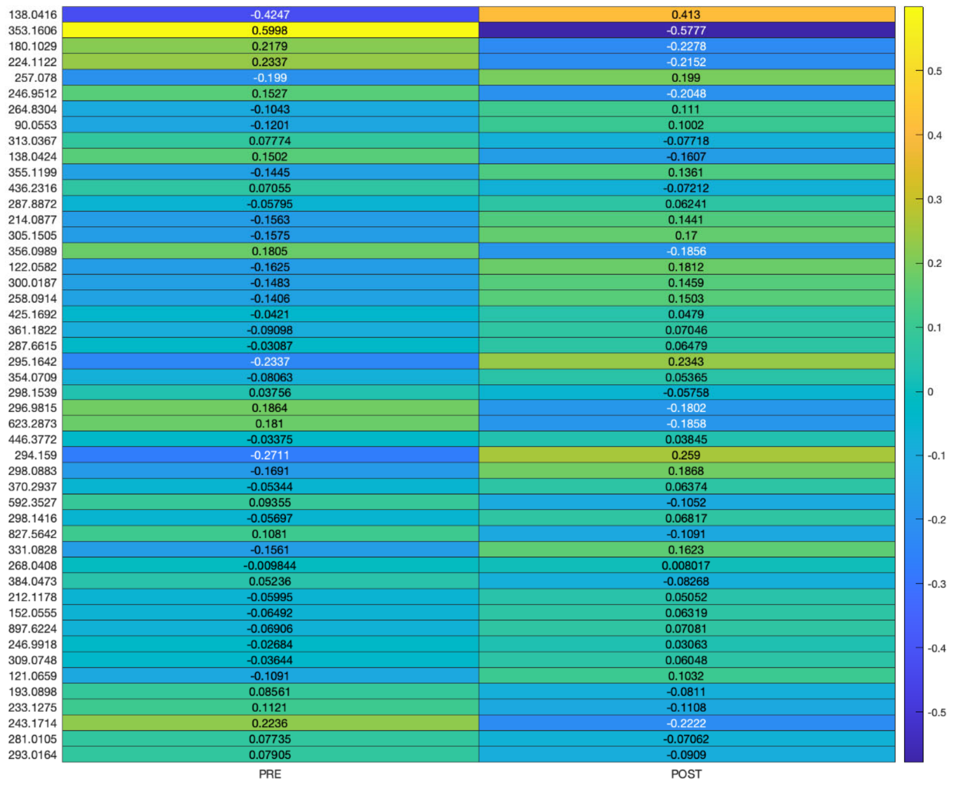

Figure 1. Heatmap matrix for the mean values of the selected features (m/z values as names) with respect to pre-post CRT status for the metabolomic dataset.

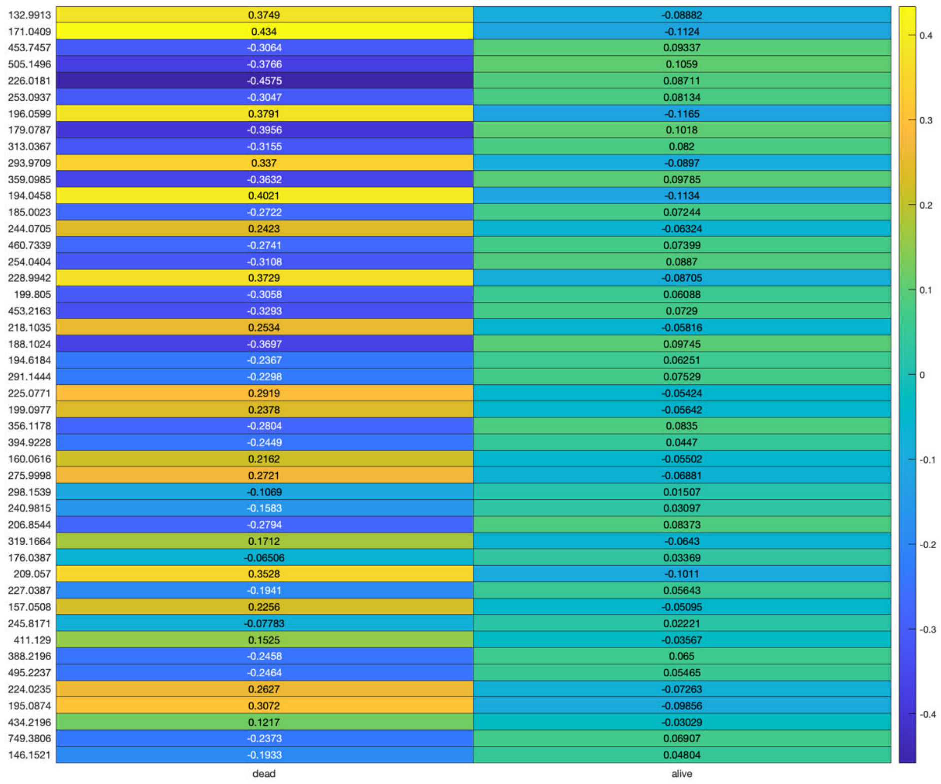

Figure 2. Heatmap matrix for the mean values of the selected features (m/z values as names) with respect to 12-month OS status for the metabolomic dataset.

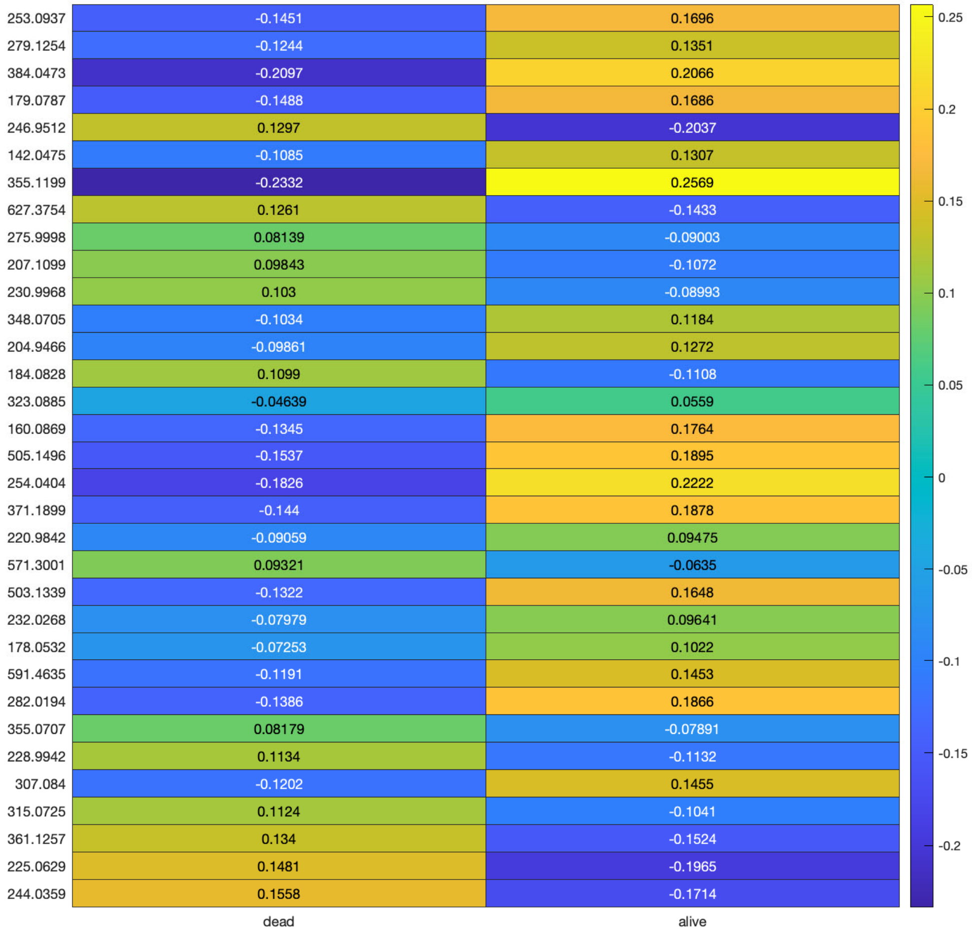

Figure 3. Heatmap matrix for the mean values of the selected features (m/z values as names) with respect to 20-month OS status for the metabolomic dataset.
